# Digital Health Interventions in the Clinical Care and Treatment of Tuberculosis and HIV in Central Ethiopia: An Initial Provider Perceptions and Acceptability Study Using the Unified Theory of Acceptance and Use of Technology Model

**DOI:** 10.4103/ijmy.ijmy_235_21

**Published:** 2022

**Authors:** Emnet Getachew, Yimtubezinash Woldeamanuel, Tsegahun Manyazewal

**Affiliations:** 1 Centre for Innovative Drug Development and Therapeutic Trials for Africa (CDT-Africa), College of Health Sciences, Addis Ababa University, Addis, Ababa,; 2 Department of Public Health, Arsi University, College of Health Science, Asella, Ethiopia

**Keywords:** Digital health, electronic health, Ethiopia technology, healthcare providers, human immunodeficiency virus, tuberculosis

## Abstract

**Background:**

Digital health technologies are emerging as promising technologies to advance clinical care. This study aimed to assess providers’ perceptions and acceptability of digital health interventions (DHIs) in the clinical care and treatment of tuberculosis (TB) and HIV in Addis Ababa, Ethiopia.

**Methods:**

This was a multi-center, facility-based, mixed-method, cross-sectional study that included 14 government health-care facilities. The participants were health-care providers (HCPs) who provide TB and HIV clinical care. Using a tool framed by the unified theory of acceptance and use of technology model, data were collected. A linear regression model was used to assess the relationship between dependent and independent variables.

**Results:**

There were 76 HCPs actively engaged in HIV/TB clinical care services in the selected 14 study sites, of whom 60 met the inclusion criteria and participated in this study. The major factors that influence HCPs’ willingness to use different technologies were educational level (β = 0.097, *t* = 3.784, *P =* 0.006), age (β = −0.227, *t* = −1.757, *P =* 0.027), work experience (β = −0.366, *t* = −2.855, *P =* 0.016). The strongest facilitator of their acceptance and the use of the digital adherence technology were perceptions of positive performance expectancy.

**Conclusion:**

Many public healthcare facilities in Addis Ababa have already begun the process of implementing various DHIs and the level of acceptability of these technologies by HCPs was found to be high.

## Introduction

Digital health interventions (DHIs) such as electronic health (eHealth), mobile health digital devices, and wireless devices are emerging as promising technologies to advance clinical care and treatment of diseases.^[1–3]^ The World Health Organization (WHO) recognizes the potential use of these technologies as discrete functionalities applied to attain health objectives.^[4]^ As a result, a wide range of DHIs has been proposed, although the impact and extent of these technologies in real-world precision medicine are ambiguous.^[5–7]^ The implementation of DHIs is made possible by several factors, including trained and qualified staff, good perception of healthcare providers (HCPs) and their willingness to use the system, and adequate infrastructure.^[8–12]^

As part of the Sustainable Development Goals, WHO, UNAIDS (The Joint United Nations Programme on HIV/AIDS) and their partners have a strategy to end tuberculosis (TB) by 2035^[13]^ and HIV by 2030^[14]^ as these are diseases of high public health concerns. According to the WHO Global TB report 2020, TB remains a worldwide threatening and one of the top 10 causes of death, with an estimated 10 million people fell ill with TB annually, and with drug-resistant TB (DR-TB) continuing to be a major global health issue.^[15]^ For HIV, according to the UNAIDS 2020 Global HIV/AIDS Report, 690 000 people died of AIDS-related illnesses in 2019 and 12.6 million of the 38 million people living with HIV were not accessing the life-saving treatment.^[16]^ Studies evaluating the effectiveness of DHIs in low-and middle-income countries are desperately needed.^[17–19]^

Ethiopia is one of the 14 triple burden countries – TB, HIV-associated TB, and multi DR-TB – and one of the 30 high-burden countries, with TB remaining one of the major causes of morbidity and mortality in the country.^[15]^ However, the Ethiopian government has been taking key measures to fight these two diseases, and as a result, Ethiopia is among the seven high-burden countries that reached the first milestone of the End TB Strategy: a 20% reduction of TB incidence between 2015 and 2020.

Understanding how theoretically promised new digital technologies to work within a specific local context is vital to understand context-sensitive implementation requirements and scale up in a wide range. Although Ethiopia’s health system has been one of the poor technologically supported sectors, new initiatives are rapidly emerging. The Ethiopian Ministry of Health recently, on 06 August 2020, launched a Digital Health Innovation and Learning Center, the first of its kind, where experts can design and validate digital health tools, synthesize and promote best practices, and scale-up innovations.^[20]^ As of the 30 April 2021 report, there were 54.7 million total telecommunication subscribers in the country.^[21]^ Mobile voice subscribers reached 52.8 million, data and internet users 25 million, fixed broadband subscribers 349,000, and fixed service subscribers 923,000. The telecom population and geographic coverage reached 95% and 85.4%, respectively, and the density reached 50%.^[21]^ The Ethiopian Health Sector Transformation Plan recognizes the need for improving digital health infrastructure to facilitate equitable access to quality healthcare for all Ethiopians.^[22]^

Several factors contribute to the success of TB/HIV programs and the perception, acceptability, and competency of the HCPs regarding the individual program is the uppermost.^[23–26]^ The objective and purpose of such new DHIs should be clear enough to all stockholders, including HCPs, for effective implementation and premier outcomes. The perceptions and acceptance of the HCPs to adopt DHIs technologies may be influenced by the overall digital health ecosystem of the country or prior experience of the HCPs using such technologies. The theoretical basis of this study is that HCPs’ perspectives are critical in determining the extent and success of implementing DHIs.

Thus, this study aimed to assess providers’ perceptions and acceptability of DHIs in the clinical care and treatment of TB and HIV in Addis Ababa, Ethiopia.

## Analytical Framework: Unified Theory of Acceptance and Use of Technology Model

**Figure 1:**
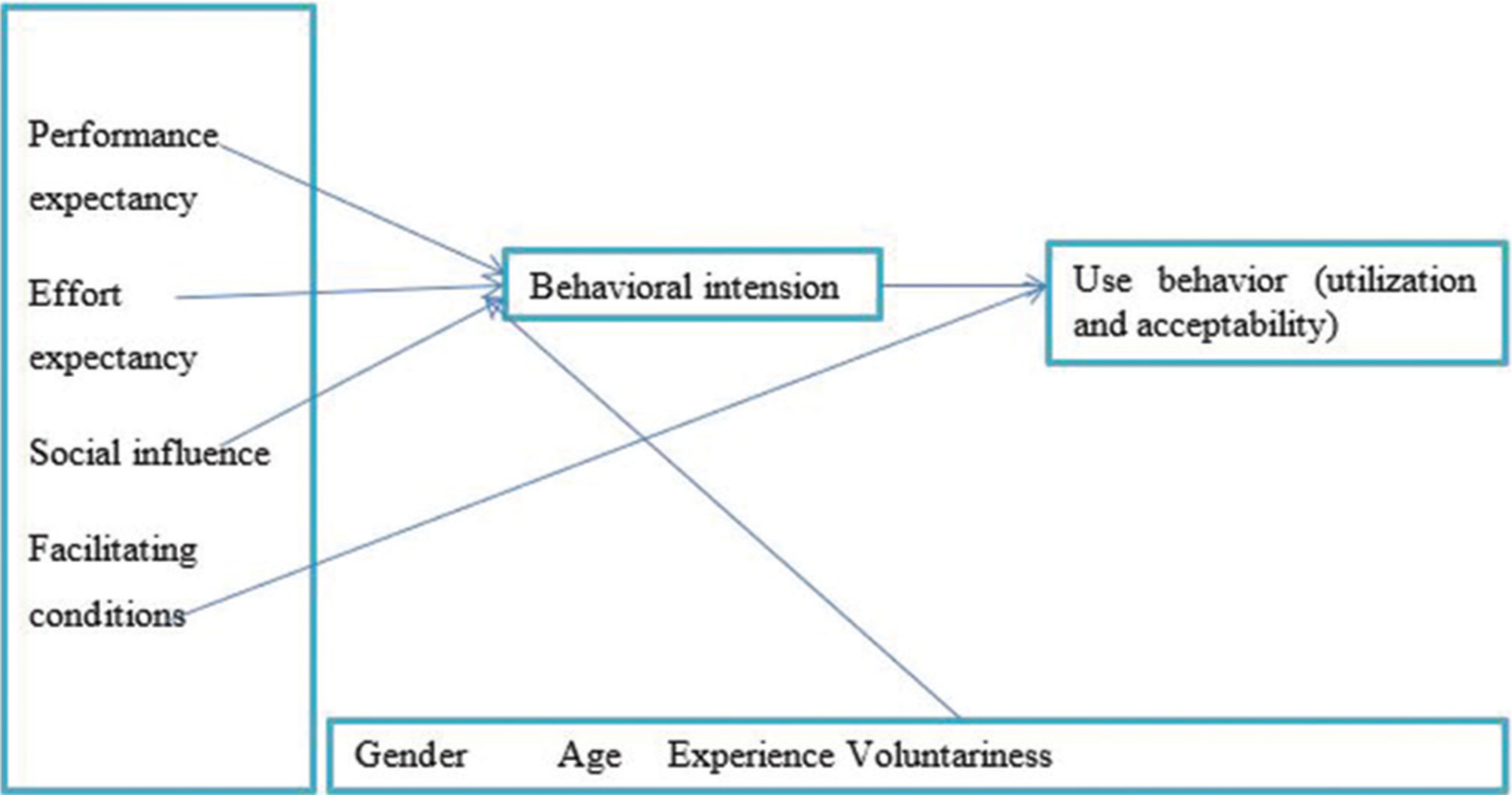
Unified theory of acceptance and use of technology framework

The unified theory of acceptance and use of technology (UTAUT) was developed to assess user acceptability of new innovative technology.^[27–29]^ It comprises multiple constructs from prior literature on technology acceptance into a single framework (acceptability of DHIs). Three of these constructs-effort expectancies, performance expectancy, and social influences are essential to comprehend the behavioral intention of individuals to utilize technology [Figure 1]. Performance expectancy refers to the perceived helpfulness and comparative benefit of technology to HCPs and the health system in general. Effort expectancy denotes the easiness of the technology to operate. Social influences mention how the perception of their patients and other HCPs in their work atmosphere.

Data from a range of other studies show that performance expectancy often has the most substantial influence on behavioral purpose to utilize technology. On the other hand, facilitating situations, the fourth construct in the UTAUT, directly influence individuals’ actual utilization of a novel technology. Facilitating circumstances include the underlying infrastructure such as human resources, power supply, Internet connection, and others to enable the use of new technology.

For HCPs, we interpreted this to hold factors such as the quality of the institutional infrastructure to assist individuals in utilizing such technology. This includes the quality of training they earned before placement of the technology and infrastructure given by the health organization to certify the proper functionality of the technologies and if any higher-level support related to the technology was offered at the time of the deployment procedure.

**Figure 2:**
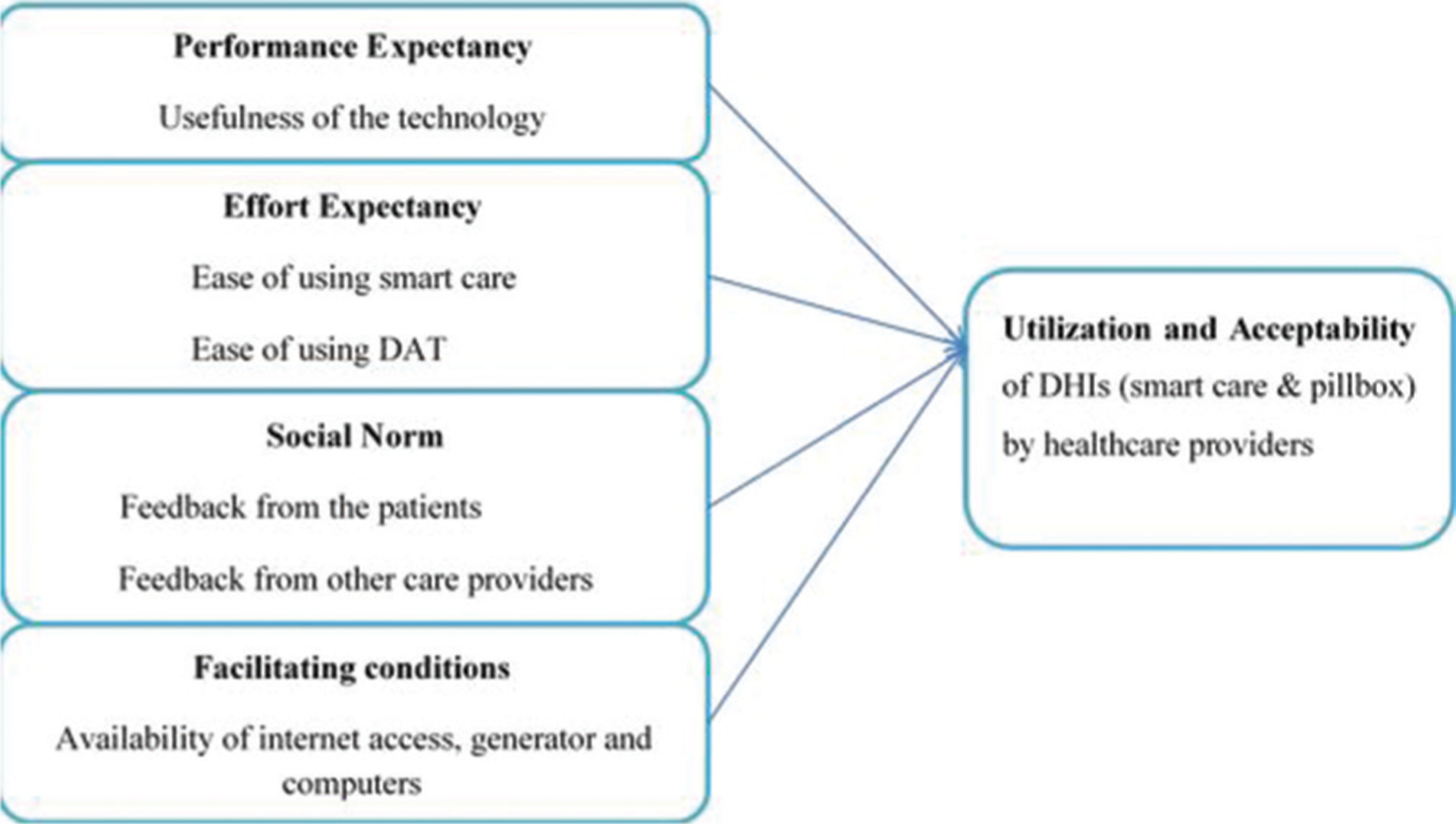
Unified theory of acceptance and use of technology model for the assessment of acceptability and perception of using digital health interventions

A central element to the acceptance of DHIs is the conservation of care providers’ time and improvement of health-care service, including improving system speed and quality of documentation [Figure 2].

## Methods

### Study setting

The study was conducted in Addis Ababa, the capital city of Ethiopia. The statistics show that the population number increases from time to time and Addis Ababa is much crowded than ever. Therefore, the people of the city are highly vulnerable to TB transmission during transportation and other social activities that put them at high risk. Studies also indicated that Addis Ababa is one of the Ethiopian cities with a large number of patients with HIV.^[30]^

There are a total of 94 health centers found in 10 sub-cities of the capital city. Of these, 10 public health centers with high TB/HIV patient flow from each sub-city were selected as indicated in Table 1.

Under the administration of Addis Ababa Health Bureau, there are 6 governmental hospitals of which 4 of them were identified as being having high TB/HIV patient flow. Accordingly, for the study purpose, we took these hospitals, i.e., Dagmawi Menelik Referral Hospital, Zewditu Memorial Hospital, Ras Desta Damtew Memorial Hospital, and Yekatit 12 Hospital.

### Study design

A facility-based mixed-method cross-sectional study was conducted in both public health centers and governmental hospitals in Addis Ababa to investigate the capacity and readiness of these facilities to incorporate various DHIs. The study was combined semi-structured interviews and a self-administered questionnaire in a mixed-methods study design. The study was conducted between January and March 2021 in Addis Ababa, Ethiopia.

### Source and study populations

- Source population: TB/HIV care providers from all healthcare facilities of Addis Ababa were the source population for the study
- Study Population: all TB/HIV care providers working in the selected health-care facilities were considered as the study population.

### Eligibility criteria

#### Inclusion criteria

- Health-care professionals were who had willing to provide informed consent
- Professionals working in selected TB and HIV clinics at the time of data collection.

#### Exclusion criteria

- Working experience on TB/HIV care for less than 6 months.

### Sample size determination

Healthcare workers were interviewed and requested to fill out self-administrated questionnaires based on purposive sampling. In the purposive sampling technique, predefined criteria were used to determine the selection of participants based on the requirements of the study objectives. Using purposive sampling, participants were selected for both questionnaires and interviews within the study cities of 10 public health centers and 4 hospitals. Accordingly, fourteen sites were sampled as part of a larger evaluation with a high TB/HIV patient load. This was incorporating all health workers meeting inclusion criteria at each site for the interview and to fill the prepared questionnaires.

As the information obtained, in health centers on average there were 8 HCPs working on TB/HIV clinics in health centers and an average of 13 HCPs in hospitals. Total HCPs who were assigned in 10 health centers and 4 hospitals were found to be 76 in number. Of these, 60 HCPs met the inclusion criteria and became the sample of this study which covered 80% of the total health-care workers as respondents from the selected healthcare facilities.

### Data collection

Data were gathered from healthcare workers using adopted questionnaires as well as semi-structured interview questions based on the adapted UTAUT model. Interview questions were used to investigate the relative advantages and simplicity of DHIs, social acceptance, and enabling factors to utilize technologies using the model. The acceptability of technology was asked only for those HCPs who held solid prior experiences of using DHIs in their health-care delivery or if they were involved in any technology utilization-related research or study.

Current technology utilization, personal perception, and acceptability of the technology in selected health facilities were investigated using interview questions and interview-based questionnaires that were replied to by the respondents.

Health workers usually work in very busy clinics, therefore; to ensure their privacy and to manage their time; the researchers used available empty office spaces. The interview was lasted for about 20 to 30 minutes and was conducted in Amharic or English depending on the interests of the respondents or the participants. The audio recording was later transcribed and translated to produce de-identified English-language transcripts.

### Epistemological approach

The epistemological approach is supposed that people construct their own understanding of fact from their varied personal experiences. According to this thought during interviews, participants were needed to draw meaning with respect to how the introduction of the new technology changed their practice and impacted the lives of their patients.

### Data processing and management

To ensure the confidentiality of participants’ data, any physical records (e. g., informed consent forms and paper-based questionnaires) were safeguarded in a locked personal cabinet, and interview recordings and transcripts were also be stored on a coded password-protected computer. To verify translation accuracy, one-quarter of English language transcripts were randomly chosen and were assessed against the original audio recordings for the correctness and completeness of the gathered data.

### Data analysis

The questionnaires and checklist were treated quantitatively, but most interview questions were analyzed qualitatively using a thematic approach. Quantitative data were analyzed using SPSS computer software, version 20 (IBM Corp. Released 2013. IBM SPSS Statistics for windows, Armonk, NY: IBM Corp).

Categorical data were expressed as frequencies and percentages. After the interviews were conducted, the responses of the respondents were written down and documented. Then, two investigators manually coded qualitative data, and a thematic approach to analyze the data was utilized. The transcription procedure was followed by coding the transcripts. Consequently, the descriptive codes were documented, and analytic codes were established. Afterward, key themes were identified and recognized. A codebook comprised both descriptive and analytical codes that related themes were utilized to guide the study’s progress. Discussion and conclusion were forwarded.

Regarding statistical analysis, we used multiple regression to assess age, sex, work experience, educational level, and department, either TB or HIV clinic influence on their inclination toward technology utilization.

The researchers reviewed transcripts of respondents to endorse the content captured whether they used accurate quotes in reporting to confirm the reliability. Moreover, this study was carried out in light of the COREQ guidelines for writing qualitative data.

### Data quality assurance

Data quality assurance includes the validity and reliability of data. Validity is the accuracy of measurement to which data truthfully represent a concept. Researchers use a validity test to ensure both dependent and independent variables have been measured accurately. Accordingly, for this particular research, the validity of the data was verified by using critical statistical tools.

A pre-test of the study questionnaires was conducted at selected health facilities on 10% of the estimated sample size to check clarity and make essential amendments to the questionnaire before its actual use. The training was provided to the data collectors to avoid hypothetical bias and confirm appropriate categorization and coding of questionnaires. The study Supervisor and the Principal Investigator verified the collected data thoroughly for completeness each day.

### Ethical considerations

The following procedures were taken to ensure ethical issues in line with the purpose of the study. Approval was attained from the Scientific and Ethics Review Committee of the Center for Innovative Drug Development and Therapeutic Trials for Africa (CDT-Africa), College of Health Sciences, Addis Ababa University on December 11/2021 with reference number CDT/1463/20. Another approval has been attained from the Ethics Committee of Addis Ababa Health bureau. An official letter was also sent to each health facility to get permission to undertake the study accordingly. Any participants, who had not willing to be included in the study, were not be forced to be part of the study.

### Patient consent

At the individual level, after a clear explanation of the purpose and importance of the study, written informed consent was obtained from all participants before they participated in the study. During the consenting process, the participants were informed that personal identifiers were omitted to ensure their privacy and confidentiality. The study units’ culture, language, beliefs, and values were considerably respected, and participants were asked permission for digital recording of the interview sessions. The study participants were informed that the study process had no intention to harm them, and there was the possibility to withdraw from the study at any time.

## Results

### Digital technology utilization status of healthcare providers

#### Sociodemographic characteristics

As indicated in Table 2, 60% of participants were females. 41.7% of them were aged between 31 and 40 years, 65% of them hold a BSc degree, 61.7% of respondents were working in an HIV clinic and 36.7% of them had more than 10 years of working experience.

#### Technology utilization

Technology utilization by HCPs was assessed. The research was conducted in 14 healthcare facilities under Addis Ababa health offices. Based on the responses of the respondents and also revealed in Table 3, 80% of HCPs used DH technologies. The HCPs from HIV clinics utilized smart care systems to keep patient data electronically and report the data to the concerned bodies and retrieve the data whenever they think it is necessary. To use such technology appropriately, the HCPs had been given some types of training but only 26% of them were satisfied with the training given to them and the majority of the health-care providers replied that the training provided was not enough to use the technology accordingly.

According to the data obtained from the respective respondents, the TB care providers in selected hospitals were found to be less familiar with technology utilization as compared with the rest of 10 selected health centers in which TB care providers likely became one part of a pilot study using Digital Adherence Technology (DAT). For such a study, they had been using the internet (Wi-Fi) and computers to follow the drug adherence of their patients. In using this new technology, the HCPs of those health facilities had been provided pertinent training.

Among the respondents, 43.3% of them confirmed that they had a favorable working environment to utilize the new technologies in their respective facilities. In addition to this, the majority of the respondents, 81.7% endorsed as being there were a lot of opportunities in the health facilities to properly implement DHIs in both health centers and public hospitals under the study.

### Acceptability and perception using digital health interventions

To assess the technology acceptability and perception of HCPs about using DHIs, an interview-based questionnaire and open-ended interview guiding questions were used. Based on the framework used, assessment has been made on the extent of technology acceptability and perceptions of HIV care providers who had solid experiences in using the smart care; which is used as a digital recording of patients’ data including card number, CD4 count, viral load and other essential information. It was observed that among the selected hospitals, unfortunately at the time of data collection only one hospital of TB clinic tried to implement a new system which helped them to record patient’s data and connected digitally with different departments such as laboratory technicians and pharmacists. Likewise, the other three hospitals had also planned to implement such technologies but were not executed yet. It was evidenced that a pilot study had been conducted in selected 10 health centers to use a digital adherence technology. The HCPs working in TB clinics were also assessed their perception and acceptability towards the digital adherence technology. Similarly, the perception and acceptability of HIV caregivers toward smart care were assessed accordingly.

The strongest facilitator of their acceptance and the use of the digital adherence technology were perceptions of positive performance expectancy (i. e., perceived usefulness). In particular, most HCPs felt that remote monitoring of medication adherence had some benefits for both patients and HCPs. During the pilot implementation of the technology, TB patients were generally dispensed 15 days of medications in the device. Patients who visited the clinic to pick up medications, which were previously required daily were likely reduced substantially. It was evidenced that the assumption of remote monitoring of adherence minimized the need for more frequent in-person monitoring. Most HCPs felt that patients benefited considerably from this reduction in required clinic visits. HCPs in the HIV clinic also described how smart care facilitated their works since they could easily retrieve patients’ data from the record so that patients’ follow-up was become easier in turn this situation likely enhanced their job satisfaction as well. Table 4 summarizes the direct speeches of the respondents.

### Willingness to use various health intervention technologies

R2 for the overall model was 54% with an adjusted R2 of 51%, a large size effect is reported by the model [Table 5].

The overall model was significant to predict care providers’ willingness to use various technologies in their facility: F (4, 54) = 15.536), *P* = 0.03 as shown by ANOVA Table 6.

**Figure 3:**
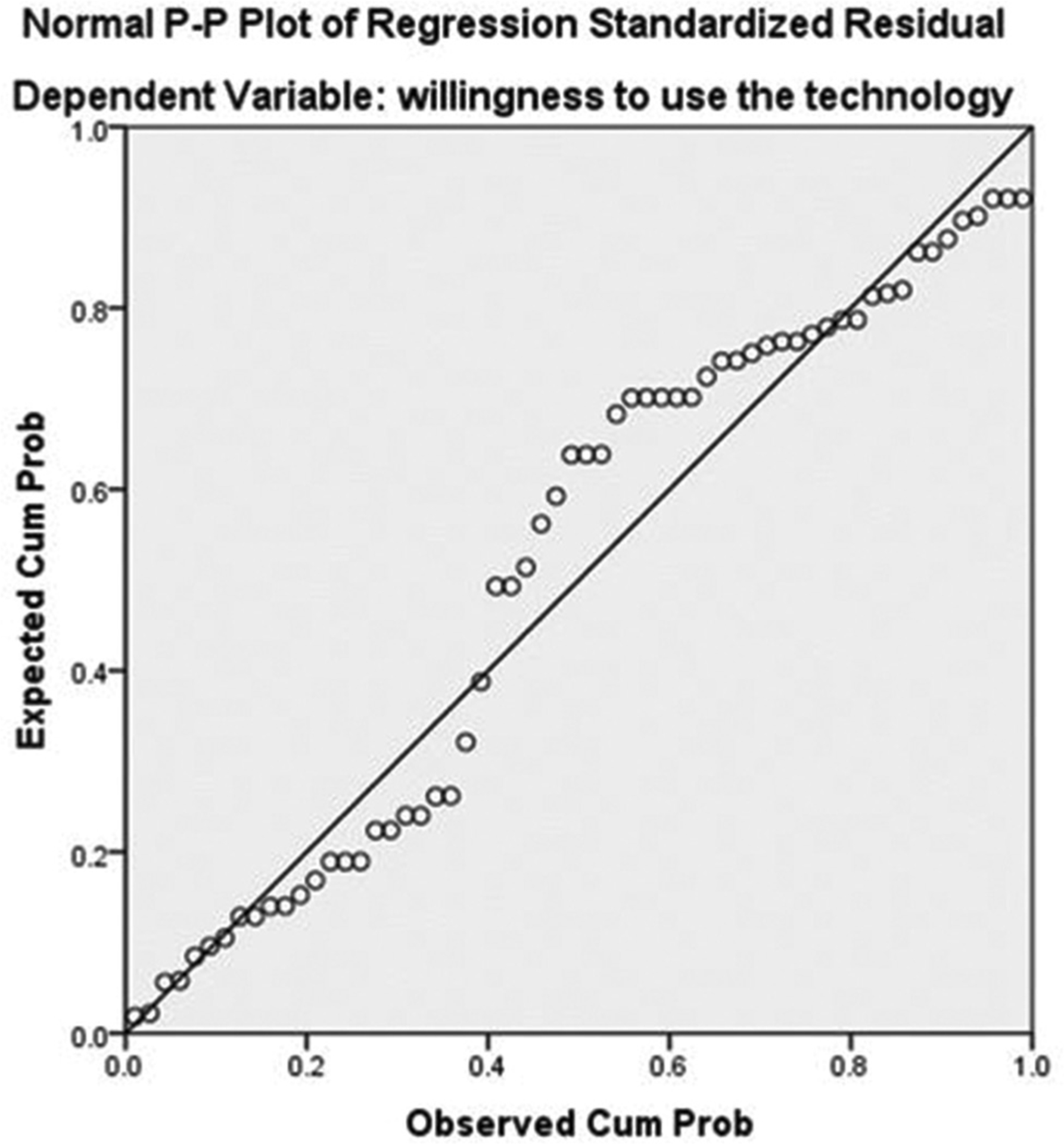
Probability plot of regression standardized residual

This study was conducted to determine if various factors can influence HCPs’ willingness to use different technologies. It was hypothesized that their sex, educational level, their department either TB or HIV clinic, age, work experience will predict their willingness toward the use of technologies. To test this hypothesis, multiple regression analysis was used. Results show that 51% of the variance can be accounted for by the five predictors, collectively, F (4, 54) = 15.536), P = 0.03. Looking at the unique individual contribution of the predictors, the result shows that educational level (β = 0.097, t = 3.784, P = 0.006), age (β = −0.227, t = −1.757, P = 0.027), Work Experience (β= −0.366, t = −2.855, P = 0.016) [Table 7]. Furthermore, the result also reveals that work experience and the age of HCPs negatively influence their willingness. Nevertheless, as the educational level of the HCPs increase, their willingness to use the technology is also increasing. However, other variables do not contribute to HCPs’ inclination towards technology utilization [Figure 3].

## Discussion

In this study, we assessed the current technology utilization by HCPs, the level of acceptance and perception toward the technology and the overall readiness of the sampled healthcare facilities to adopt and implement different new technologies. Among the respondents, 80% of them have ever used different technologies in their respective healthcare facilities to support health-care delivery. This finding is similar to other studies conducted elsewhere in comparable settings.^[31,32]^ According to the respondents, the Internet access of the healthcare facilities was found to be 100%. However, this data had some discrepancies regarding Internet access among healthcare workers. Thus, HIV care providers had more access to use the Internet relative to HCPs working in TB clinics. On the other hand, few Participants in this study confirmed that they used the Internet for regular medical/professional updates. A possible reason mentioned was that the poor computer hardware and very slow Internet connection at the selected health-care facilities.

The majority of HCPs included in the study had better access to computers at their respective healthcare facilities. This finding is important since, better access to such technology among HCPs working in health-care facilities, advance their healthcare service. In fact lack of access to technology mainly to computers is assumed as a major barrier to the adoption of ICTs in their respective workplaces which is similar to other results of studies.^[33]^ Besides, our study found that HCPs with access to computers believed that they would personally benefit from the implementation of a digital health system. As a result, they would gain the necessary skills for the implementation of such a system of technology in more innovative ways.

A previous study conducted among HCPs in some primary care practices found that providers that used computers often were more eager regarding the technology and that providers who already have been using computers believed that they had the skills for adapting to newly implemented technologies.^[33]^ Another study conducted in London showed that the current ICT utilization among HCPs acts as a facilitator for the acceptance and use of eHealth technologies. Those HCPs, who used the computer at the center daily, believed that they had the necessary skills for the implementation of such a system as compared to those who never used the computers at the center. This corroborates or verifies with the findings from another study conducted in a primary healthcare center setting.

Some studies found that the deficit of basic and refreshment training on computers and eHealth among health professionals is the possible hindrance factor for the utilization of DHIs in health-care facilities that might lead to undesirable patient health outcomes.^[34]^ This is actually reported in the same fashion by the current study in that health professionals who had ever trained for the DHIs were more likely to be willing to use the DHIs than their counterparts as supported by previous studies.^[34]^ This is also actually in line with the existing fact that training can change the knowledge, attitude, and skills of health professionals on computer systems and as a result increase commitment to use the DHIs in particular and the other technologies in general. Thus, before the actual launching of such kind of program, it is mandatory to assess the existing level of knowledge, acceptance, and utilization habits of those HCPs in the facilities.

Drawing from the UTAUT model, we found that a digital adherence monitoring intervention by TB care providers and smart care is largely acceptable for supporting TB and HIV care delivery in the selected health-care facilities. Overall, the key factor for acceptability appeared to be perceived usefulness; for TB caregivers, although the electronic adherence monitor was only initially intended to monitor adherence, it was also beneficial in creating a sense of being “cared for” and a sense of fear of “being caught” not adhering, both of which inspired participants to take medications to maintain their ongoing relationships with the study staffs. SMS text messages not only reminded patients to take the medication in time but also enabled social supporters to provide medication-taking-related support.

For HIV care providers the key factor for acceptability also appeared to be perceived usefulness; care providers were able to retrieve any patient-related data including patients’ cards, date of appointment, CD4 count, viral load, and other pertinent information. Reliable and timely health information is the foundation of health systems action where information and communication technology initiatives such as Smart Care helps electronically keep documents and enhance the decision-making process. However, it is sometimes not available when required because of poor managerial priority, budget allocation, and support. This was truly explained by the current study by the fact that managerial support, guidelines, and access to strong signal internet were independent determinants for acceptability and utilization of the system. This result is similar to that of the study conducted in Ethiopia.^[34]^ This might be explained by the fact that when managerial support is in place, more resources including working manuals would be allocated to the implementation, undoubtedly health professionals become motivated and eager to use the technology.

## Conclusion

Many public healthcare facilities in Addis Ababa have already begun the process of implementing various DHIs and e-Health systems for TB/HIV services and the level of acceptability of these technologies by HCPs was found to be high. However, most of the available digital health technologies in the facilities were utilized without reliable DHIs/eHealth regulatory policy in place. Thus, there is a critical need for reliable DHIs/e-Health regulatory policies and some improvement is needed in DHIs/e-Health strategic planning (core readiness). There should be a prior need assessment and proper training given to HCPs to properly adopt and implement new DHIs in healthcare facilities.

### Limitation of the study

This study was undergone with selected subjects with the limited number of participants and selected governmental health-care facilities in Addis Ababa. Therefore, a replication or transfer of the result of this study to other parts of Ethiopia, particularly for the rural areas and the private health-care environment, should consider the potential differences resulting from varying cultural, socioeconomic, infrastructural, and political backgrounds healthcare is a much-institutionalized industry. The same carefulness must be executed when applying this study’s result in other developing countries and worldwide.

## Figures and Tables

**Table 1:** Included public health centers from each sub-city

Name of health center	Sub-city
Addis Raey health center	Addis ketema
Akaki health center	Akaki kality
Kebena health center	Arada
Goro health center	Bole
Adisu Gebeya health center	Gulele
Kazanchis health center	Kirkos
Alem Bank health center	Kolfe
Teklehaymanot health	Lideta
Woreda 2 health center	Nifasilk lafto
Woreda 13 health center	Yeka

**Table 2:** Sociodemographic characteristics

	*n* (%)
Total	60 (100)
Gender	
Male	24 (40)
Female	36 (60)
Age	
18–30	16 (26.7)
31–40	25 (41.7)
41–50	15 (25)
Above 51	4 (6.7)
Educational level	
College diploma	10 (16.7)
BSc	39 (65)
MSc	11 (18.3)
Department	
TB room	23 (38.3)
HIV room	37 (61.7)
Work experience	
<1 year	2 (3.3)
2–5	16 (26.7)
6–9	20 (33.3)
Above 10	22 (36.7)

TB: Tuberculosis, HIV: Human immunodeficiency virus

**Table 3:** Responses of leading questions by the respondents

Leading questions	Percent of cases
Q1^a^	
HCPs heard of DHIs	48.3
HCPs with smart phone	85.0
Willingness to use various technologies in the facility	90
Computer access in the health-care facility	55.0
HCPs having different online training	11.7
Experience using EMR	61.7
Experience in using any other technologies for TB/HIV patients	80.0
The relative advantage of technology	75.0
The simplicity of the technology	70.0
Related training that would help to implement such technology	68.3
Adequacy of the training	26.7
Favorable environment or infrastructure	43.3
Challenges to use DHIs in the facility?	86.7
Opportunities in the facility to adopt new DHIs?	81.7

a Question 1. HCPs: Health-care providers, DHIs: Digital health interventions, EMR: Electronic medical record, TB: Tuberculosis, HIV: Human immunodeficiency virus

**Table 4:** Direct speech of the respondents

Response
Performance expectancy “We can retrieve patient data from electronically recorded documents easily as compared with manual work documentation; besides the systems do have numerous advantages that simplify service delivery and decision-making process”. HCPs from HIV clinic “I think smart care has made our work much easier. Before there were a lot of missing data of the patients but currently any important information can be easily accessed by the care providers”. HCPs from HIV clinic “The digital adherence technology reduced the workload and increased our job satisfaction which allows us to dedicate more time to other tasks”. HCPs from TB clinic “Previously we were supposed to give therapy on a daily basis (i.e., DOT). But now, the patients come (to the clinic) every 15 days so that our work pressure has been reduced consequently”. HCPs from TB clinic “I am not sure whether they have taken the medication or not thus i prefer DOT”. HCPs from TB clinic Effort expectancy “We took two weeks training on how to use the smart care and consequently it likely becomes very easy to work with it as a result of its utilization”. HCPs from HIV clinic “I found the drug adherence technology is easy to use. I have not yet faced any difficulties so far to use it.” HCPs from TB clinic “With a simple orientation I was given, I found the task was very difficult for I have no previous experience on using such technologies, but currently I can use the device more easily after sometimes of exposure.” HCPs from TB clinic Social norm “Patients now need not travel long distances spending their money and time to collect their drugs every day. Most of our TB patients come from distant villages. Now, they feel comfortable with being supported by these technologies.” HCPs from TB clinic “Previously most patients come to the clinic with lost cards and it was difficult to manage such issues but currently since the patient’s card number is registered electronically as a result of this the problem is being solved. Therefore, the patients are happy with the new technology” HCPs from HIV clinic “The good thing about the digital adherence technology is that it does not carry any messages on TB [on the outside of the box], so there is no stigma attached to it. This helps the patients to carry it freely”. HCPs from TB clinic Facilitating conditions “Here in our offices, we have enough computers so we can perform our work more easily than before”. HCPs from HIV clinic “Even if there is Wi-Fi access in the health facility, the signal is a bit weak to work with it efficiently”. HCPs from TB clinic “We had two weeks of training on smart care to implement it; besides, the management members are very supportive. There are also enough number computers, Wi-Fi access and generator; so, this would facilitate our work”. HCPs from HIV clinic “In our room, there are no computers and the one that we had used previously is not yet maintained; so, we have borrowed some computers from another room and this makes our works very difficult for there is a very limited number of computers to use in the health facility.” HCPs from TB clinic

HCPs: Healthcare providers, HIV: Human immunodeficiency virus, TB: Tuberculosis

**Table 5:** Model summary

Model	*R*	*R* ^2^	Adjusted *R*^2^	SE of the estimate	Change statistics				
					*R*^2^ change	*F* change	Df1	Df2	Significant *F* change
1	0.436^a^	0.540	0.515	0.461	0.590	2.536	5	54	0.030^b^

a Predictors: Constant, work experience, department, educational level, sex, age,

b Dependent variable: Willingness to use the technology. SE: Standard error

**Table 6:** ANOVA table

Model	Sum of squares	Df	Mean square	*F*	Significant
1					
Regression	2.697	4	0.539	15.536	0.030^b^
Residual	11.486	54	0.213		
Total	14.183	59			

a Dependent variable: Willingness to use the technology,

b Predictors: Constant, work experience, department, educational level, sex, age

**Table 7:** Table of coefficients

Model	Unstandardized coefficients		Standardized coefficients	*T*	Significant	Correlations			Collinearity statistics	
	*B*	SE	Beta			Zero-order	Partial	Part	Tolerance	VIF
Constant	1.611	0.346		4.655	0.000					
Education	0.080	0.102	0.097	3.784	0.006	0.152	0.106	0.096	0.974	1.027
Sex	0.055	0.127	0.055	0.433	0.666^a^	0.056	0.059	0.053	0.916	1.092
Department	0.056	0.073	0.100	0.767	0.085	−0.051	0.104	0.094	0.875	1.143
Age	−0.227	0.129	−0.227	−1.757	0.027^a^	−0.199	−0.233	−0.215	0.896	1.116
Experience	−0.203	0.071	−0.366	−2.855	0.016^a^	−0.362	−0.362	−0.350	0.913	1.095

a Dependent variable: Willingness to use the technology. SE: Standard error, VIF: Variance inflation factor

## References

[1] van Buchem MM, Boosman H, Bauer MP, Kant IM, Cammel SA, Steyerberg EW. The digital scribe in clinical practice: A scoping review and research agenda. NPJ Digit Med 2021;4:57. 33772070 10.1038/s41746-021-00432-5 PMC7997964

[2] Briganti G, Le Moine O. Artificial intelligence in medicine: Today and tomorrow. Front Med (Lausanne) 2020;7:27. 32118012 10.3389/fmed.2020.00027 PMC7012990

[3] Agarwal P, Gordon D, Griffith J, Kithulegoda N, Witteman HO, Sacha Bhatia R, Assessing the quality of mobile applications in chronic disease management: A scoping review. NPJ Digit Med 2021;4:46. 33692488 10.1038/s41746-021-00410-x PMC7946941

[4] World Health Organization (WHO). Global Strategy on Digital Health 2020–2025. Genea, Switzerland; WHO; 2020. Available from: https://www.who.int/docs/defaultsource/documents/gs4dhdaa2a9f352b0445bafbc79ca799dce4d.pdf. [Last accessed on 2020 Aug 02].

[5] Gunasekeran DV, Tseng RM, Tham YC, Wong TY. Applications of digital health for public health responses to COVID-19: A systematic scoping review of artificial intelligence, telehealth and related technologies. NPJ Digit Med 2021;4:40. 33637833 10.1038/s41746-021-00412-9 PMC7910557

[6] Capobianco E, Iacoviello L, de Gaetano G, Donati MB. Editorial: Trends in Digital Medicine. Front Med (Lausanne) 2020;7:116. doi:10.3389/fmed.2020.00116. 32309285 PMC7145957

[7] Grande D, Luna Marti X, Feuerstein-Simon R, Merchant RM, Asch DA, Lewson A, Health policy and privacy challenges associated with digital technology. JAMA Netw Open 2020;3:e208285. 32644138 10.1001/jamanetworkopen.2020.8285 PMC7348687

[8] Whitelaw S, Pellegrini DM, Mamas MA, Cowie M, Van Spall HG. Barriers and facilitators of the uptake of digital health technology in cardiovascular care: A systematic scoping review. Eur Heart J Digit Health 2021;2:62–74. 34048508 10.1093/ehjdh/ztab005 PMC8139413

[9] Mogessie YG, Ntacyabukura B, Mengesha DT, Musa MB, Wangari MC, Claude N, Digital health and COVID-19: Challenges of use and implementation in sub-Saharan Africa. Pan Afr Med J 2021;38:240. 34046143 10.11604/pamj.2021.38.240.27948 PMC8140728

[10] Meier L, Tippenhauer K, Sariyar M. Decentralized digital health services caught between the pressure for innovation and the burden of regulations. Stud Health Technol Inform 2021;281:1046–50. 34042838 10.3233/SHTI210344

[11] Wu A, Scult MA, Barnes ED, Betancourt JA, Falk A, Gunning FM. Smartphone apps for depression and anxiety: A systematic review and meta-analysis of techniques to increase engagement. NPJ Digit Med 2021;4:20. 33574573 10.1038/s41746-021-00386-8 PMC7878769

[12] Manyazewal T, Woldeamanuel Y, Blumberg HM, Fekadu A, Marconi VC. The potential use of digital health technologies in the African context: A systematic review of evidence from Ethiopia. NPJ Digit Med 2021;4:125. 34404895 10.1038/s41746-021-00487-4 PMC8371011

[13] World Health Organization (WHO). End TB strategy. Geneva, Switzerlands; WHO; 2014. Available from: https://www.who.int/tb/strategy/End_TB_Strategy.pdf?ua=1. [Last accessed on 2020 Aug 02].

[14] The Joint United Nations Programme on HIV/AIDS (UNAIDS). Global AIDS Monitoring 2021. Geneva, Switzerlands; UNITAID; 2021. Available from: https://www.unaids.org/en/resources/documents/2020/global-aids-monitoring-guidelines. [Last accessed on 2020 Aug 02].

[15] World Health Organization (WHO). Global Tuberculosis Report 2020. Geneva, Switzerland: WHO; 2020.

[16] UNAIDS. Global HIV Statistics 2020. Geneva, Switzerland: UNAIDS; 2020. Available from: https://www.unaids.org/sites/default/files/media_asset/UNAIDS_FactSheet_en.pdf. [Last accessed on 2020 Aug 02].

[17] Zaidi HA, Wells CD. Digital health technologies and adherence to tuberculosis treatment. Bull World Health Organ 2021;99:323–323A. 33958817 10.2471/BLT.21.286021 PMC8061661

[18] Manyazewal T, Woldeamanuel Y, Blumberg HM, Fekadu A, Marconi VC. The fight to end tuberculosis must not be forgotten in the COVID-19 outbreak. Nat Med 2020;26:811–2. 32398877 10.1038/s41591-020-0917-1 PMC7570951

[19] Holst C, Sukums F, Ngowi B, Diep LM, Kebede TA, Noll J, Digital health intervention to increase health knowledge related to diseases of high public health concern in Iringa, Tanzania: Protocol for a mixed methods study. JMIR Res Protoc 2021;10:e25128. 33885369 10.2196/25128 PMC8103301

[20] John Snow, Inc. (JSI). Ethiopia Launches Digital Health Innovation and Learning Center. August 6^th^, 2020 | NEWS. Available from: https://www.jsi.com/ethiopia-launches-digital-health-innovation-and-learning-center/. [Last accessed on 2020 Aug 02].

[21] Ethiotelecom. Statistics. Addis Ababa, Ethiopia, Ethiotelecom; April, 2021. Available from: https://www.ethiotelecom.et/. [Last accessed on 2020 Aug 02].

[22] Ethiopia Ministry of Health. The Ethiopian Health Sector Transformation Plan II. Addis Ababa, Ethiopia: Ethiopian Ministry of Health; 2019. Available from: https://www.moh.gov.et/ejcc/am/node/152. [Last accessed on 2020 Aug 02].

[23] Subbaraman R, de Mondesert L, Musiimenta A, Pai M, Mayer KH, Thomas BE, Digital adherence technologies for the management of tuberculosis therapy: Mapping the landscape and research priorities. BMJ Glob Health 2018;3:e001018. 10.1136/bmjgh-2018-001018 PMC6195152 30364330

[24] Thomas DS, Daly K, Nyanza EC, Ngallaba SE, Bull S. Health worker acceptability of an mHealth platform to facilitate the prevention of mother-to-child transmission of HIV in Tanzania. Digit Health 2020;6:2055207620905409. 32076575 10.1177/2055207620905409 PMC7003162

[25] Manyazewal T, Marinucci F, Belay G, Tesfaye A, Kebede A, Tadesse Y, Implementation and evaluation of a blended learning course on tuberculosis for front-line health care professionals. Am J Clin Pathol 2017;147:285–91. 28395055 10.1093/ajcp/aqx002

[26] Mussie KM, Gradmann C, Manyazewal T. Bridging the gap between policy and practice: A qualitative analysis of providers’ field experiences tinkering with directly observed therapy in patients with drug-resistant tuberculosis in Addis Ababa, Ethiopia. BMJ Open 2020;10:e035272. 10.1136/bmjopen-2019-035272 PMC7304814 32554739

[27] Williams MD, Rana NP, Dwivedi YK. The Unified Theory of Acceptance and Use of Technology (UTAUT): A literature review. J Enterp Inform Manag 2015;28:443–88.

[28] Heinsch M, Wyllie J, Carlson J, Wells H, Tickner C, Kay-Lambkin F. Theories informing eHealth implementation: Systematic review and typology classification. J Med Internet Res 2021;23:e18500. 34057427 10.2196/18500 PMC8204232

[29] Ammenwerth E Technology acceptance models in health informatics: TAM and UTAUT. Stud Health Technol Inform 2019;263:64–71. 31411153 10.3233/SHTI190111

[30] Kibret GD, Ferede A, Leshargie CT, Wagnew F, Ketema DB, Alebel A. Trends and spatial distributions of HIV prevalence in Ethiopia. Infect Dis Poverty 2019;8:90. 31623689 10.1186/s40249-019-0594-9 PMC6796490

[31] Shiferaw KB, Mengiste SA, Gullslett MK, Zeleke AA, Tilahun B, Tebeje T, Healthcare providers’ acceptance of telemedicine and preference of modalities during COVID-19 pandemics in a low-resource setting: An extended UTAUT model. PLoS One 2021;16:e0250220. 33886625 10.1371/journal.pone.0250220 PMC8061916

[32] Jones C, Miguel-Cruz A, Brémault-Phillips S. Technology acceptance and usability of the BrainFx SCREEN in Canadian Military Members and Veterans With Posttraumatic Stress Disorder and Mild Traumatic Brain Injury: Mixed methods UTAUT study. JMIR Rehabil Assist Technol 2021;8:e26078. 33983125 10.2196/26078 PMC8160786

[33] Saleh S, Khodor R, Alameddine M, Baroud M. Readiness of healthcare providers for eHealth: The case from primary healthcare centers in Lebanon. BMC Health Serv Res 2016;16:644. 27832788 10.1186/s12913-016-1896-2 PMC5105285

[34] Berihun B, Atnafu DD, Sitotaw G. Willingness to use Electronic Medical Record (EMR) system in healthcare facilities of Bahir Dar City, Northwest Ethiopia. Biomed Res Int 2020;2020:3827328. 32908886 10.1155/2020/3827328 PMC7471823

